# Numerical Simulation and Optimization of 4-Component LDG Separation in the Steelmaking Industry Using Polysulfone Hollow Fiber Membranes

**DOI:** 10.3390/membranes12010097

**Published:** 2022-01-17

**Authors:** Jong-Yeol Jeon, Bo-Ryoung Park, Jeong-Hoon Kim

**Affiliations:** C1 Gas & Carbon Convergent Research Center, Korea Research Institute of Chemical Technology, 141 Gajeongro, Yuseong-gu, Daejeon 34114, Korea; jjyok@krict.re.kr (J.-Y.J.); brpark@krict.re.kr (B.-R.P.)

**Keywords:** CO_2_ recovery, Lintz Donawiz converter gas, polysulfone hollow fiber, membrane separation, multicomponent gas, finite element model, permeance simulation

## Abstract

A general finite element model and a new solution method were developed to simulate the permeances of Lintz Donawiz converter gas (LDG) components and the performance of a polysulfone membrane separation unit. The permeances at eight bars of CO, N_2_, and H_2_ in LDG simulated using the developed model equations employing the experimental mixed gas data were obtained by controlling the finite element numbers and comparing them with pure gas permeation data. At the optimal finite element numbers (*s* = 15, *n* = 1), the gas permeances under the mixed-gas condition were 6.3% (CO), 3.9% (N_2_), and 7.2% (H_2_) larger than those of the pure gases, On the other hand, the mixed-gas permeance of CO_2_ was 4.5% smaller than that of pure gas. These differences were attributed to the plasticization phenomenon of the polysulfone membrane used by CO_2_. The newly adopted solution method for the stiff nonlinear model functions enabled the simulation of the performance (in terms of gas recovery, concentration, and flow rate) of the first-stage membrane within two seconds under most gas flow conditions. The performance of a first-stage membrane unit separating LDG could be predicted by the developed model with a small error of <2.1%. These model and solution methods could be utilized effectively for simulating gas permeances of the membrane that is plasticized severely by the permeating gas and the separation performance of two- or multi-stage membrane processes.

## 1. Introduction

Emissions of greenhouse gases into the atmosphere are still growing despite the enormous efforts to mitigate them. In Korea, CO_2_ emissions from the combustion of fossil fuels increased from 594 to 704 million tons from 2010 to 2018 [1]. The Intergovernmental Panel on Climate Change has reported that anthropogenic CO_2_ emissions should be reduced to net-zero by 2050 relative to 2010 levels to limit global warming to 1.5 °C above pre-industrial levels [2]. We focused on the separation and utilization of CO_2_ and CO in the Lintz Donawiz converter gas (LDG, 64% CO, 18% CO_2_, 16% N_2_, and 2% H_2_) in the steelmaking industry while a variety of attempts have been devoted toward the reduction of CO_2_ emissions. In Korea, LDG is combusted to generate electricity with other byproduct gases in the steel industry, such as coke oven gas (COG) and blast furnace gas (BFG), causing a large amount of CO_2_ emissions. The main components of LDG are CO and CO_2_. Therefore, CO_2_ emissions to the atmosphere can be reduced by recovering CO and CO_2_ and utilizing them as raw materials to produce various chemical products.

Several separation technologies, such as cryogenic distillation, absorption, and adsorption, have been considered for industrial applications to separate CO_2_ and CO concentrations from the LDG mixed gases. CO_2_ separation using separation technologies has been extensively studied and commercialized. On the other hand, CO separation using adsorbents or absorbents using copper transition metals was developed and commercialized in 2000, including the chemical absorption process by R.C. Costello & Associate Inc., USA (Cosorb or Copure process) and pressure swing adsorption (PSA) process by Kobe Steel Co., Japan and Pioneer Technology Inc., China. However, these methods require high separation energy in the processes due to the strong binding of the adsorbents or adsorbents with CO. Moreover, their unsteady operation behavior due to low CO/CO_2_ selectivity of Cu(I) instability and impurity poisoning from hydrogen sulfide or water vapor has severely limited their scale-up and wide commercial applications.

To solve these problems, we have designed a new hybrid-type CO purification process employing membrane separation to remove CO_2_ and H_2_ selectively in LDG and recover high-purity CO from N_2_ residues using a typical PSA, which is fed to membrane modules to separate it into a residue stream containing mostly CO and N_2_ and a permeate stream consisting of CO_2_ and H_2_. Then, the residue CO/N_2_ mixture is sent to a PSA or chemical absorption process unit to separate it into high purity CO above 99% and the residue N_2_. Ag(I)-based facilitated transport membrane processes could also be an alternative to the typical PSA to separate the residue CO/N_2_ mixture. To accomplish this task effectively, a high-performance membrane that selectively permeates CO_2_ and H_2_ in LDG, the process simulation, and design techniques must be developed in which the membrane separation of LDG uses a hollow fiber membrane. This includes the mathematical modeling and simulation required for the scale-up of the membrane separation process.

Since the early 1970s, various mathematical models and solution methods for membrane gas separation processes have been reported in the literature [3,4,5,6,7,8,9,10,11,12,13,14,15,16,17,18,19,20,21,22,23,24,25,26,27]. The models can be classified into two categories according to their equation forms: differential and finite element models. The differential models, where the governing equations of motion are expressed in the form of differential equations, can be conveniently applied to binary systems because all equations can be solved analytically. The differential models are still useful for multi-component concurrent systems where feed and permeate gases flow concurrently because their governing equations can be solved step by step by numerical integrations without any assumptions. However, in the case of multicomponent countercurrent systems, integrating differential equations numerically iteratively (trial-and-error shooting method) [28,29,30,31], the flow rates and concentrations of each component in the permeate leaving membrane module are very cumbersome and time-consuming because the nonlinear equations of membrane permeation are very stiff. To avoid integration, various methods that convert the differential equations into algebraic forms, such as series approximation [32,33,34], discretization [35], and orthogonal collocation [36,37] approaches, have been suggested.

The derived governing equations of the finite element models have algebraic forms. These models regarded the membrane as being composed of a number of finite elements having the same area in which both permeate, and residue side gases are in a perfectly mixed state. Therefore, the gas concentrations are all the same at any position within a finite element. The governing equations obtained from the mass balance around the finite elements are composed only of algebraic variables. These models can be applied effectively to hollow fiber membrane modules where gas mixing occurs compared to differential models that assume, in general, plug-flow on both permeate and residue sides. With the extent of gas mixing, the number of finite elements can be adjusted identically or differently on both sides, which is an advantage not found in other models. In a hollow fiber membrane module, if gas mixing takes place, it would be more likely on the shell side than on the bore side because the shell side has more free space for gas to move. In this case, it is desirable that the bore and shell sides have different finite element numbers from each other.

In this study, we investigated the general finite element model with an arbitrary ratio *n* of the bore to the shell side finite element number to determine the value of *n* at which the model agrees best with the experimental data. A set of derived algebraic equations were solved by the NEQNF subroutine in the IMSL library, which is a method of solving a system of nonlinear equations using the modified Powell hybrid algorithm and a finite-difference approximation to Jacobian. Using the subroutine, we can solve nonlinear equations much faster and easier compared to other methods suggested in previous studies. Based on the model, two Fortran programs have been developed for the simulation, one for calculating the permeances of LDG mixed gas using the experimental data obtained under one experimental condition and the other for predicting the performance of a used membrane (flow rates and concentrations of the LDG components in residue and permeate) using the permeance data calculated using the former program.

Generally, the permeances of mixed gas used in simulations of membrane separation processes are those of pure gases [29,30,36], those calculated using linear cross-flow models [38,39], or those obtained by experiments at a low stage-cut of less than 1% [40,41,42] with the assumption that the concentrations of feed and residue gases are the same or at a stage-cut of less than 20% [10,43,44,45]. The latter permeances were calculated using the logarithmic mean concentrations of feed and residue gases as residue-side concentrations. However, these are all approximate values that are not acquired under the real operating conditions of the membrane modules. Therefore, in this study, we calculated the permeances of the LDG mixed gas using the data obtained under real operating conditions of the membrane module.

## 2. Materials and Methods

### 2.1. Mathematical Model

#### 2.1.1. Bore Side Feed

**Figure 1:**
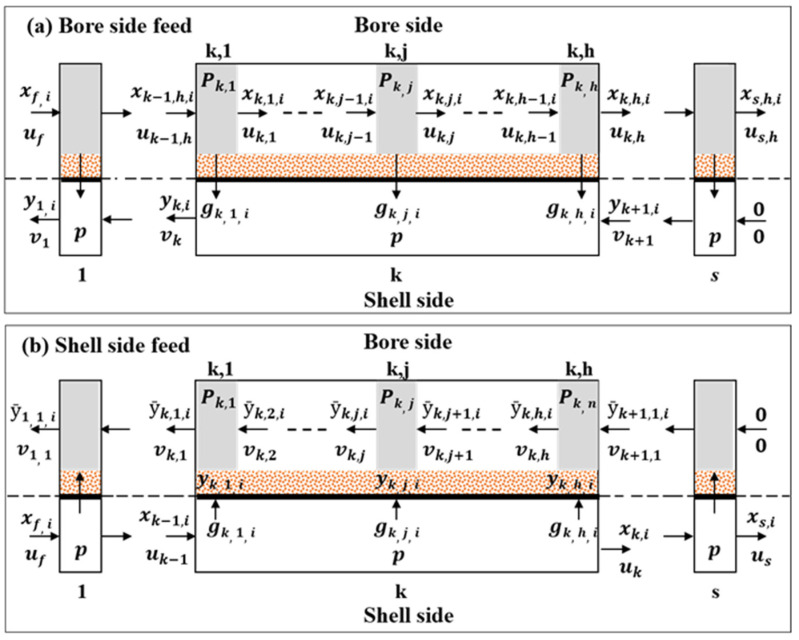
Schematic of a general finite element model for asymmetric hollow fiber membrane. (**a**) Bore side feed and (**b**) shell-side feed.

Katoh et al. [46] developed a finite element model for a hollow fiber membrane module called the “tank-in-series model.” Here, the value of *n* was fixed at 2. We deal with the most general case of this model, where *n* is not specified at a certain value for an asymmetric hollow fiber membrane. For each finite element of the shell side, the countercurrent hollow fiber membrane is divided into finite elements on the shell side and *h* finite elements on the bore side, as shown in Figure 1. For convenience, the *k*-th element is verified.

The following equations are obtained by mass balance and fluid dynamics for the *k*-th element of the bore side feed mode (Figure 1a) under isothermal and steady-state conditions if the membrane does not deform under pressure and the pressure drop of the bore side follows the Hagen–Poiseuille law.

(1) The permeation rate of the *i*-component in the *k*,*j*-th element of the bore side is

(1) $$g_{k,j,i}=Q_{i}A_{j}\left(P_{k,j}x_{k,j,i}-py_{k,i}\right).$$

Summing $g_{k,j,i}$ for all the components (*c*) gives

(2) $$G_{k,j}=\sum_{i=1}^{c}g_{k,j,i}$$

The summation of Equation (1) for the *i*-component on the bore side is:

(3) $$GC_{k,i}=\sum_{j=1}^{h}g_{k,j,i}$$

The summation of the permeation rates of all the components in the bore side is

(4) $$GT_{k}=\sum_{i=1}^{c}GC_{k,i}$$

(2) Component and total mass balances
  - The *k,j*-th element on the bore side is expressed as
    (5) $$u_{k,j-1}x_{k,j-1,i}-u_{k,j}x_{k,j,i}-g_{k,j,i}=0$$
    (6) $$u_{k,j-1}-u_{k,j}-G_{k,j}=0$$
  - Meanwhile, the *k,j*-th element in the permeate side is given by
    (7) $$v_{k+1}y_{k+1,i}-v_{k}y_{k,i}+GC_{k,i}=0$$
    (8) $$v_{k+1}-v_{k}+GT_{k}=0$$

The pressure drop between the (*k,j* − 1)-th and *k,j*-th elements is

(9) $$P_{k,j-1}-P_{k,j}=\frac{128u_{k,j}\mu_{m}L_{j}}{\pi D_{o}^{2}},$$

where *μ_m_* is the viscosity of the residue side gas calculated using the Wike equation [47].

#### 2.1.2. Shell Side Feed

The lengthwise and backmixing of gases is very restrictive within the porous supporting layer of the asymmetric hollow fiber membrane. Therefore, gases can pass through the layer in the cross-flow type. When gases flow in this manner, a cross-flow model can be set up for the shell side feed mode. However, for the bore side feed, this model is difficult to realize mathematically. The concentrations of permeate are different at the wall of the selective layer, in which high permeability gases permeate selectively, and in the bulk permeate stream of the bore side. If the concentration of the *i*-component is y_i_ at the selective layer wall and ${\bar{y}}_{i}$ in the permeate bulk stream (Figure 1b) the governing equations for the cross-flow model are as follows:

(1) The permeation rates in the *k*-th element:

(10) $$g_{k,j,i}=Q_{i}A_{j}\left(px_{k,i}-P_{k,j}y_{k,j,i}\right)$$

(11) $$G_{k,j}=\sum_{i=1}^{c}g_{k,j,i}$$

(12) $$GC_{k,i}=\sum_{j=1}^{h}g_{k,j,i}$$

(13) $$GT_{k}=\sum_{i=1}^{c}GC_{k,i}$$

(2) The component and total mass balances in the *k*-th and *k,j*-th elements:

(14) $$v_{k,j+1}{\bar{y}}_{k,j+1,i}-v_{k,j}{\bar{y}}_{k,j,i}+g_{k,j,i}=0$$

(15) $$v_{k,j+1}-v_{k,j}+G_{k,j}=0$$

(16) $$u_{k-1}x_{k-1,i}-u_{k}x_{k,i}-GC_{k,i}=0$$

(17) $$u_{k-1}-u_{k}-GT_{k}=0$$

(3) The concentration y*_k,j,i_* at the *k,j*-th element:

(18) $$y_{k,j,i}=\frac{g_{k,j,i}}{G_{k,j}}=\frac{Q_{i}A_{j}\left(px_{k,i}-P_{k,j}y_{k,j,i}\right)}{\sum_{i=1}^{c}Q_{i}A_{j}\left(px_{k,i}-P_{k,j}y_{k,j,i}\right)}$$

(4) The pressure drop between the (*k,j* − 1)-th and *k,j*-th elements:

(19) $$P_{k,j+1}-P_{k,j}=\frac{128v_{k,j}\mu_{m}L_{j}}{\pi D_{o}^{2}},$$

where *μ_m_* is the viscosity of the permeate side gas mixture.

### 2.2. Solution of the Model Equation

Katoh et al. [46] solved the nonlinear equations of their finite element model using the relaxation method, which has been applied to distillation processes. On the other hand, Coker et al. [48,49] used the Thomas algorithm after converting a set of flow rate equations into a tridiagonal matrix form. However, as mentioned previously, nonlinear equations can be solved more conveniently and rapidly by the NEQNF subroutine than by other methods. The subroutine requires only rough initial values for all variables to start the calculation. Given the initial values, it finds the final values of all the variables satisfying a given error tolerance, iteratively modifying the Jacobians of coded equations. Before coding, nonlinear equations must be rearranged such that one side is equal to zero, as shown in Equations (5)–(8). Subsequently, they must be set to variables *F*(*x*), which then becomes an error in each equation. The NEQNF subroutine provides the sum of *F*(*x*)^2^ instead of *F*(*x*), which can have negative real values. From $\sum F{\left(x\right)}^{2}$, the root mean square error of the NEQNF method can be calculated as

(20) $$\sqrt{\frac{\sum F{\left(x\right)}^{2}}{x},}$$

where *x* is the total number of coded equations.

For the bore side feed operation, there are a total of 11 unknown variables, except for the feed compositions, feed rate, bore side pressure, and permeances of the gas components, when *s* = 1, *n* = 1, and *c* = 4. In the case of the shell side feed, there are four additional unknown variables (i.e., the permeate concentrations at the wall of the selective layer y_i_). We can find the values of the unknown variables (concentrations, flow rate, and pressure) by solving 11 and 15 equations from Equations (5)–(9) and (14)–(19) for the bore side feed and shell side feed, respectively. In contrast, if there are experimental data for the concentrations and flow rates of the residue and permeate leaving the membrane, we can calculate the mixed gas permeances of the four components. The number of unknown variables is five at *s* = 1 and *n* =1. Therefore, the permeances can be calculated using five equations from Equations (9) and (5) or Equation (7) for the bore-side feed. For the general case (i.e., *s* = *s*, *h* = *h*, and *c* = 4), there are a total of (6s*h* + 5*s*) and (6*sh* − 1) variables for the performance-simulating model that predicts concentrations and flow rates and permeance-simulating model for the bore side feed, respectively. The performance and gas permeance of the membrane can be obtained using as many equations from Equations (5)–(9) as the variables.

### 2.3. Experimental

**Figure 2:**
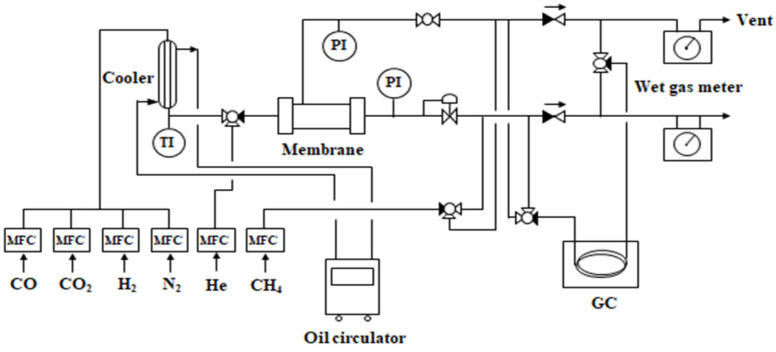
Schematic diagram of the one-stage membrane separation process for LDG separation (PI: pressure indicator; TI: temperature indicator; GC: gas chromatography; MFC: mass flow controller).

Figure 2 shows a schematic of the membrane separation unit installed for LDG separation. To develop the model, polysulfone (PS) hollow fiber modules (Synopex Inc., Ltd., Hwaseong, Korea) were used as the membrane, whose specifications are listed in Table 1. In the LDG model gas, the gas mixture (64% CO, 18% CO_2_, 16% N_2_, and 2% H_2_ by volume) was made by flowing pure gases with mass flow controllers and fed to the bore side of the membrane module. The mass flow controllers were recalibrated with a standard wet gas meter (Shinagawa Co., Tokyo, Japan) before use. The membrane module was maintained at 20 °C by a heat exchanger that controlled the temperature of the feed gas and an air conditioner installed in the equipment room. The gas lines were connected to ensure that the membrane operated in countercurrent mode. The flow rates and concentrations of the residue and permeate leaving the membrane were measured with wet gas meters and an Agilent 6890 GC (Agilent Technologies, Inc., Santa Clara, CA, USA) using CH_4_ as an external standard gas. For precise measures of flow rates, the wet gas meters were installed in a constant temperature oven kept at 3 °C to suppress as much as possible the vaporization of their working fluid (water) into the process gas streams, which increased the gas flow rates. After the analysis, all waste gases were burned in a flare stack.

## 3. Results and Discussion

Glassy polymers such as PS and PI are plasticized by penetrant CO_2_ [38,50,51,52,53,54,55]. The permeabilities of other gases increase if a membrane is swollen by CO_2_. Therefore, their permeances differ when permeating with CO_2_ and as pure gases. The permeances of pure gases are not suitable for simulating CO_2_-containing mixed-gas separation processes. Here, we calculated the permeances of LDG mixed gas using the model with experimental data obtained at one feed flow rate condition. Then, the effectiveness of the approach was verified through experiments under other conditions. Two Fortran programs were prepared, one for calculating the gas permeances and the other for simulating the performance of the PS membrane.

Table 2 presents the experimental results at a feed flow rate of 10 L/min, feed pressure of eight bars, and permeate pressure of one bar.

Using these data, the permeances of the LDG components were calculated using the permeance-simulating program. Table 3 summarizes the simulated results while varying the finite element numbers of the bore and shell sides. In addition, the permeances and selectivities of the pure gases are also included in the table for comparison. The results show that the permeances of the more permeable gas CO_2_ are lower than those of pure CO_2_ in the mixed gas state, while those of the less permeable gases CO and N_2_ are slightly larger than those of the pure gases. In addition, the CO_2_ permeances decreased by 0.2-10.3% at the investigated finite element numbers, while those of CO and N_2_ increased by 5.9–9.67% and 3.5–4.7%, respectively, in comparison to those of pure gases. Considering that the permeance differences between the pure and mixed gases are not large, the plasticization effect is not significant in the used PS membrane. In our CO recovery process, the CO_2_/CO and CO/N_2_ selectivities are important properties. The CO selectivity for CO_2_ and N_2_ increased when permeated with CO_2_. Accordingly, the actual CO recovery is lower than predicted with the selectivity data of their pure gases. This means that gas mixing occurs over a wider range in the membrane as the number of finite elements decreases. In contrast, the gas flow pattern in the membrane module becomes closer to the plug-flow type as the number of finite elements increases. In general, the performance of the membrane is better in the plug-flow mode than in the mixed-flow mode. According to the simulation results, the permeances of the more permeable gases CO_2_ and H_2_ decrease with an increase in the finite element number, and those of the less permeable gases CO and N_2_ exhibit the opposite trend. This is due to the input performance data required for the simulation to be fixed at the values listed in Table 2. The performance of the membrane would increase and the permeances of more permeable gases should decrease if the number of finite elements increases to satisfy the input performance data.

Table 4 presents the experimental results at feed rates of 5, 20, and 30 L/min, a feed pressure of 8 bars, and a permeate pressure of one bar. We investigated the finite numbers at which the model best fits the experimental data presented in Table 3 and Table 4. In the LDG separation process, the most important performance measures of the membrane are CO recovery (the percentage of the CO flow rate in the residue to the flow rate in the feed gas) and CO and CO_2_ concentrations in the residue. Table 5 shows the simulation errors with respect to the experimental data for the performance measures of the PS membrane with the variation in feed flow rates. The simulations were performed with permeance data of *s* = 15 and *n* = 1, as shown in Table 3. Based on the calculated percent errors, the root mean square percent errors (RMSPE) for the experimental results of the four feed flow rates were calculated as follows:

(21) $$\sqrt{\frac{\sum {\left(percent\;error\right)}^{2}}{4}.}$$

Table 5 lists the results. In this manner, the RMSPEs were calculated at other values of s and n. We can see that the model agrees very well with the experimental data. The RMSPEs for CO recovery and CO and CO_2_ concentrations in the residue were 1.42%, 0.16%, and 2.12%, respectively, in the feed flow rate range of 5–30 L/min, respectively. The RMSPE for the CO_2_ concentration in the residue was relatively large because the absolute values of the CO_2_ concentrations in the residue were small. When the gas concentration is small, a small experimental error can significantly affect the percent error.

**Figure 3:**
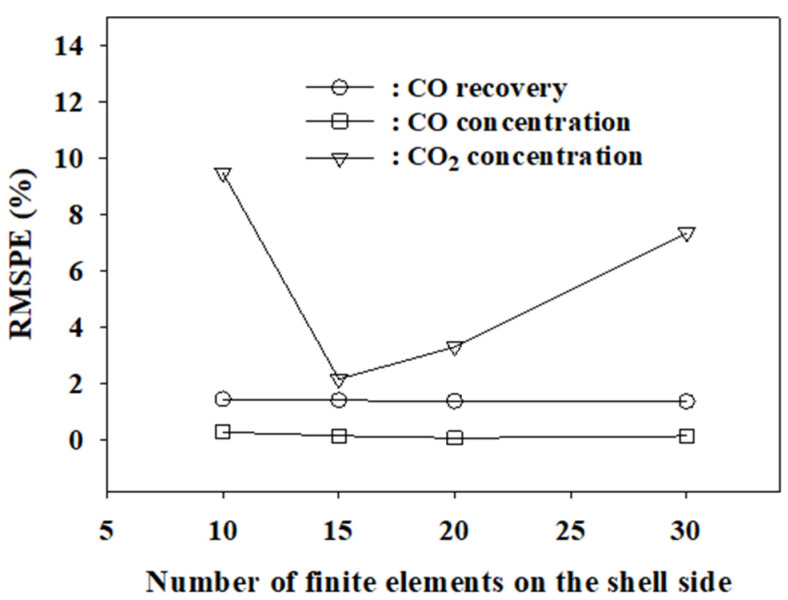
RMSPE for the major performance indicators of the membrane according to *s* value at *n* = 1.

**Figure 4:**
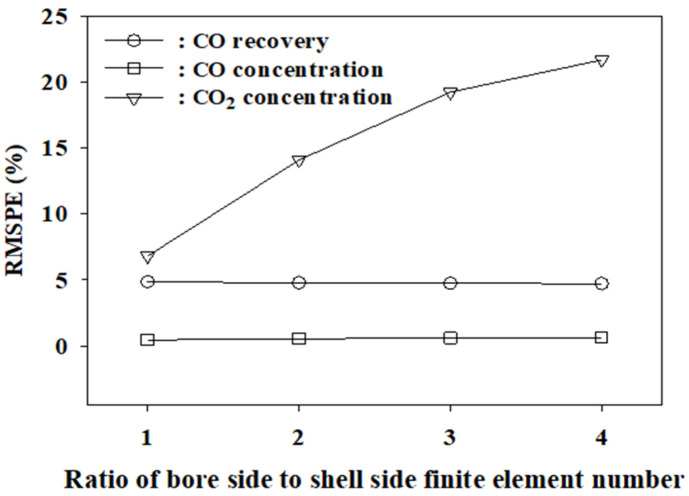
RMSPE for the major performance indicators with the variation of *n* at *s* = 15.

**Figure 5:**
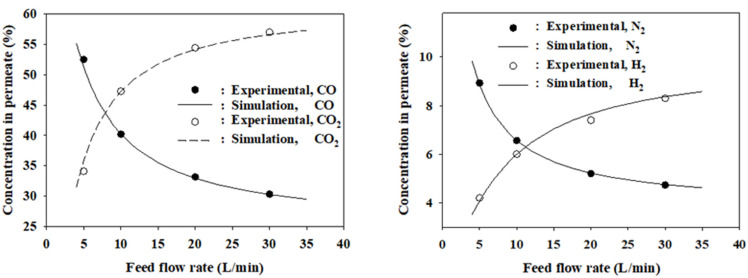
Comparison of simulated and experimentally measured concentrations of the components in the permeate.

Figure 3 shows the graphs of RMSPE against *s* at *n* = 1. The variation of RMSPEs for CO recovery and CO concentration in the residue was very small in the simulated range of *s* = 10–30. However, the RMSPE for the concentration of the more permeable gas CO_2_ varies considerably according to the *s* value. The lowest RMSPE for the concentration of CO_2_ in the residue was attained at the number of shell side finite elements *s* = 15. Overall, at *s* = 15, the RMSPEs for the major performance measures of the membrane were the lowest. As shown in Figure 4, at *s* = 15, the RMSPE for the CO_2_ concentration increases significantly with an increase in *n*, while the RMSPEs for CO recovery and CO concentration are nearly constant. From these results, the optimal values of *s* and *n*, where the RMSPEs are the lowest, are 15 and 1, respectively. In conclusion, the finite element model agrees best with the experimental data when there are 15 finite elements on both the shell and bore sides. It was expected that the model would fit better at an *n* value greater than one because the shell side has more free space for gases to move. However, it fits best at the same finite number of 15 on both sides. The RMSPEs of the three major performance measures of the membrane were below 2.1% for the four feed flow rates, as shown in Table 5. The agreement between the model and the experimental data is also excellent for the concentrations of the components in the permeate, as shown in Figure 5.

Table A1 (Appendix A) presents the RMSPEs for the major performance measures calculated similarly to what is outlined above using the permeance data of pure gases at various *s*. The RMSPEs for the CO concentrations in the residues were below 2.24% in the range of *s* = 2–60. Although the RMSPE for the CO_2_ concentrations in the residue was the lowest (2.26%) at *s* = 15, it significantly increased up to 171% at other values of s. The RMSPEs for CO recovery are nearly constant at approximately 3.06%. These figures are not as large as the root mean square errors, but the low values result from the low percentage errors at high feed flow rates. The percent errors between the model and experimental data at *s* = 15 and *n* = 1 are −5.63, −1.55, −1.22, and −1.21% at feed flow rates of 5, 10, 20, and 30 L/min, respectively. Therefore, we cannot accurately predict the performance of the membrane at low feed flow rates using the permeance data of pure gases. In conclusion, the fluctuation in RMSPE for the CO_2_ concentration in the residue was severe when pure gas permeances were used in the simulation, and the CO recovery could not be predicted with low error at low feed flow rates, indicating that pure gas permeances are inappropriate as permeance data for simulating the performance of a glassy polymer membrane.

**Figure 6:**
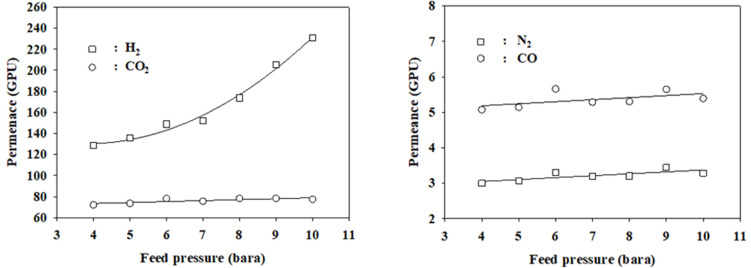
Permeances of the mixed gas components with varying feed pressures (1 GPU = 7.5 × 10^−12^ m^3^/m^2^.s.Pa).

In general, the permeability of gas increases as the pressure increases due to the increase in the diffusivity through the membrane [56,57,58,59]. The permeances of CO_2_, CO, and N_2_ in the mixed-gas state, which were calculated from the experimental data using the model at the optimal finite element numbers (*s* = 15, *n* = 1) in Table 3, increased marginally and linearly with the feed pressure in the range of 4–10 bars, as shown in Figure 6. However, the permeance of H_2_ increases exponentially. Any regression equations obtained from the data in Figure 6 can be incorporated into the model to predict the performance of the membrane over a wide pressure range. The simulated permeances at 8 bars of CO, N_2_, CO_2_, and H_2_ were 5.3, 3.2, and 173.7 GPU, respectively. These results are 6.3%, 3.9%, and 7.2% larger than the permeance of each pure gas, respectively. On the other hand, the permeance (78.3 GPU) of CO_2_ in LDG was decreased 4.5% compared to that (84.0 GPU) in pure gas.

**Figure 7:**
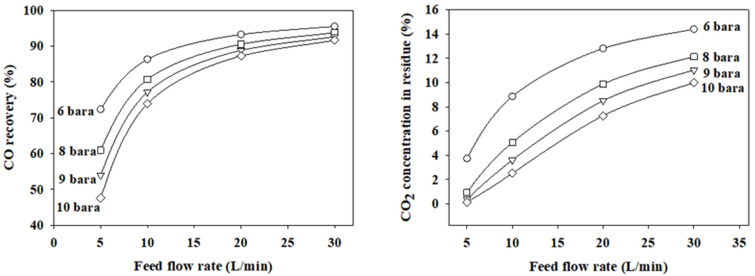
Simulated CO recoveries and CO_2_ concentrations in the residue based on the feed flow rate and feed pressure at a permeate pressure 1 bar.

Figure 7 shows the simulated CO recoveries and CO_2_ concentrations in the residue as a function of feed flow rates at the four feed pressures. Simulations were conducted using the optimal finite element.

For the one-stage membrane process. The feed pressure was investigated below 10 bars due to the high energy cost of gas compression. We observed that the CO recovery and CO_2_ concentration in the residue moved in the same direction at a feed pressure of 6–10 bars. The CO recovery and CO_2_ concentration become lower as the feed pressure becomes higher. Therefore, there is no condition that can simultaneously satisfy a high CO recovery of >95% and a low CO_2_ concentration of less than 1% at feed pressures below 10 bars. The maximum CO recovery was approximately 61% when the condition of satisfying CO_2_ concentration was less than 1% at a feed pressure of eight bars and a feed flow rate of 5 L/min.

**Figure 8:**
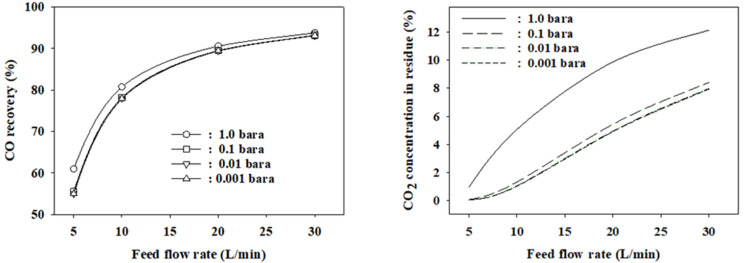
Permeate side-vacuuming effect on the CO recovery and CO_2_ concentrations in the residue.

**Figure 9:**
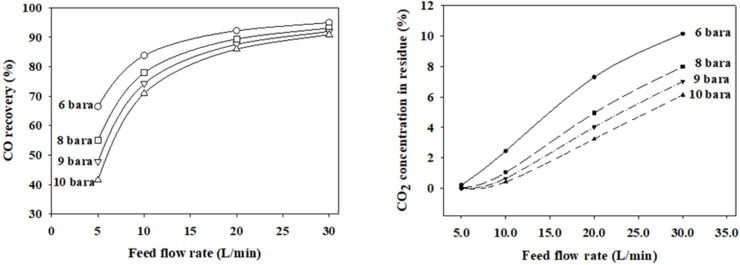
CO recovery and CO_2_ concentration in the residue with the variation of feed flow rate and feed pressure at the permeate side pressure of 0.1 bar.

The permeation rate through the membrane can be increased by drawing a vacuum on the permeate side because the permeation driving force increases by reducing the permeate side partial pressure. Simulations were carried out at the optimal finite element numbers for the vacuum operation of the permeate side in the range of 0.001–0.1 bars at a feed pressure of eight bars to determine how much the performance would improve. Compared to the atmospheric operation, the CO recovery decreased by 5.4–0.8% and the CO_2_ concentration in the residue decreased by 0.9–4.2% at feed flow rates of 5–30 L/min and the permeate side pressure of 0.1 bar. However, at lower vacuum pressures, they did not further decrease, as shown in Figure 8. Considering the energy cost of vacuum operation, the optimal pressure is 0.1 bar if the permeate side is operated in the vacuum mode. In the LDG separation process to recover CO of chemical grade, the most important figure of merit is CO purity. In the membrane separation step of the membrane-PSA hybrid process, the desired CO_2_ concentration level in the residue was below 1%. The maximum CO recovery satisfying this figure of merit is approximately 78% at a feed pressure of eight bar and a feed flow rate of 10 L/min when the permeate side is operated in the vacuum mode. Compared with the atmospheric operation of the permeate side, the CO recovery and the LDG separation rate increased from 61% to 78% and 5 to 10 L/min, respectively, by vacuuming of the permeate side. Figure 9 shows the simulation results at a permeate pressure of 0.1 bar with varying feed pressures. The CO_2_ concentration in the residue decreased with increasing feed pressure. The maximum feed flow rate satisfying a CO_2_ concentration of less than 1% is 12.6 L/min at a feed pressure of 10 bars. Compared to the simulation results at a feed pressure of 8 bars, the LDG separation rate increased by 2.6 L/min. However, the CO recovery decreased when the feed pressure was increased to 10 bars and the feed pressure at the permeate side pressure is 0.1 bar.

One of our research objectives for the membrane separation of LDG is to recover CO gas with less than 1% CO_2_ with a recovery of >95%. However, the objective cannot be attained in the one-stage membrane process, even if the feed pressure can be raised to 50 bars, indicating that a multi-stage process is required to achieve this goal. At present, a two-stage membrane separation process is under development.

**Figure 10:**
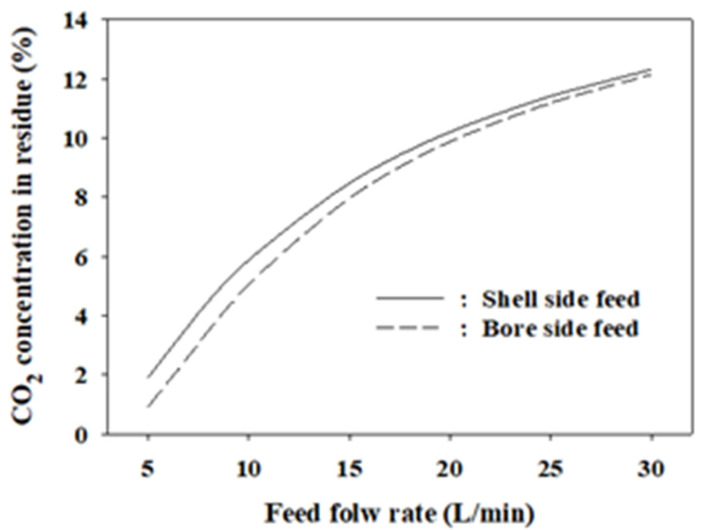
Comparison of the CO_2_ concentrations in the residue in the bore side and shell side feed modes.

In an asymmetric hollow fiber membrane, the shell side feed mode can raise the feed gas pressure to a higher level than in the bore side feed operation. However, with a short length of the membrane module, the performance is lower than that of the bore side feed operation due to the non-ideal gas flow pattern formed from entering and exiting the membrane module in the vertical direction. In addition, the dead volume between the tube sheets of the hollow fibers and gas line fittings affects the performance of a short-length membrane. As previously mentioned, gases can flow in a cross-flow pattern in an asymmetric hollow fiber membrane because of the porous support. Based on this flow, asymmetric models have been developed in the literature, although there are many cases where symmetric models still fit better [27,60]. Based on the simulation results, it appears that cross-flow and concentration polarization do not occur in the PS membrane module used herein. However, the shell side feed mode was simulated using the cross-flow model developed in Section 2.2 under the assumption that the flow occurs to determine the extent to which the cross-flow affects the performance. Figure 10 presented the comparison of the CO_2_ concentrations in the residue in the shell side and bore side feed modes simulated under the same conditions (i.e., feed pressure of 8 bars, permeate pressure of 1 bar, *s* = 15, and *n* = 1). Although the CO_2_ concentration in the shell side feed mode was higher than that in the bore side feed mode, the difference was less than 1% in the feed flow range of 5–30 L/min. In the case of CO recovery, the difference was small enough that it can be practically ignored. The gas follow patterns in the used membrane do not substantially affect the major performance measures for LDG separation.

One of the advantages of the finite element model is its ability to simulate various types of membrane modules and flow patterns (i.e., flat, hollow fiber, and spiral wound membranes, and mixed- and plug-flow membrane modules). One flat membrane module in which the gases are perfectly mixed can be simulated with the values of *s* and *n* as one. On the other hand, the plug-flow mode is realized when the finite element number is very large. The spiral wound membranes can be simulated using the cross-flow model described in Section 2.2.

**Figure 11:**
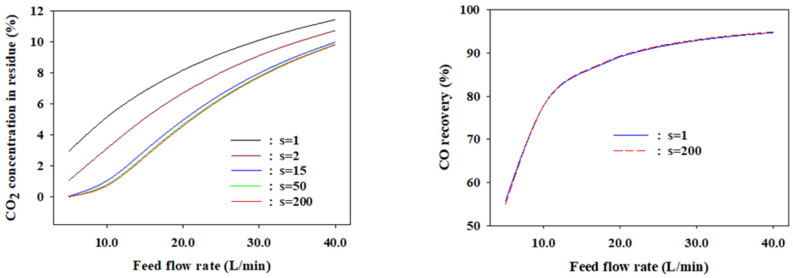
Effect of the shell side finite element number on the CO_2_ concentration in the residue and CO recovery.

Figure 11 shows the simulation results based on the finite element number of the shell side at a feed pressure of eight bars, permeate pressure of one bar, and *n* = 1. The CO_2_ concentration in the residue decreases as the finite elements increases from 1, which corresponds to a flat membrane, to 200. However, the rate of decline slows significantly after *s* = 15, and there are no noticeable changes from *s* = 100. The maximum difference in the CO_2_ concentrations between *s* = 1 (flat membrane) and *s* = 200 (plug flow membrane) was 5.1% at a feed rate of 10 L/min. The CO_2_ concentrations at the optimal finite number (*s* = 15) of the LDG separation are depicted with a blue line in Figure 11. The length per finite element is 1.6 cm and the active length of the membrane module is 24 cm. The finite element model assumes that the perfect mixing of gases occurs inside the zone of 1.6-cm length. Although perfect mixing occurs at every length of 1.6 cm, it is apparent that the CO_2_ concentrations are close to those of the plug flow membrane (*s* = 200). The differences are only 0.03–0.3% in the feed flow rate range of 5–40 L/min. Meanwhile, the recovery of the less permeable CO gas was insensitive to the finite element number. The values at *s* = 1 and 200 were almost the same.

The numbers of model equations required to obtain the data in Figure 11 based on the finite element number are presented in Table A2 (Appendix A). The CPU run times taken to solve these equations, $\sum F^{2}$ and the root-mean-square errors calculated using Equation (20) are listed in the table. The CPU run times were obtained using the Fortran command “call cpu_time()” on an Intel Core™ 3.3 GHz personal computer. The solution method for the model equations using the NEQNF subroutine is powerful and effective. At the best finite element number, it took only 0.02 s within a root mean square error of 8.4 × 10^−19^ to solve 110 equations.

## 4. Conclusions

Predicting the performance of a membrane process separating a multicomponent gas mixture containing CO_2_, which is a glassy polymer-plasticizing gas, using the permeance data of pure gases or by approximation methods is not a good approach. The permeance data of mixed gas is more accurate when measured under real operating conditions of the membrane. In this study, a general finite element model whose element numbers can be adjusted freely in both bore and shell sides was developed to simulate the permeances of LDG mixed gas and the performance of the PS membrane module. Moreover, the permeances of the LDG component gases were calculated using the model at various finite element numbers and the experimental performance data (component gas concentrations and total flow rates at the bore and shell side outlets) obtained at one feed flow rate. At the optimal finite element numbers (*s* = 15, *n* = 1), the simulated permeances at eight bars of CO, N_2_, CO_2_, and H_2_ were 5.3, 3.2, and 173.7 GPU, respectively, when the finite element number was 15. These results are 6.3%, 3.9%, and 7.2% larger than the permeance of each pure gas, respectively. On the other hand, the permeance (78.3 GPU) of CO_2_ in LDG was decreased 4.5% compared to that (84.0 GPU) in pure gas.

The differences between the pure and mixed gas permeances were due to the plasticizing effect of CO_2_ on the glassy polymer polysulfone. The permeance difference was the most remarkable in the case of H_2_, which has the smallest molecule size among the gases. There are many industrial gases in which high concentrations of H_2_ and CO_2_ are present. The representative gases are synthetic gas and many industrial byproducts such as COG, Finex gas, and petrochemical byproducts. Moreover, the pure gas permeance data could not be used when we designed a membrane process for H_2_ separation from these mixed gases. In this case, our model can provide more precise information on the permeance.

Then, we can obtain the performance data of the PS membrane at arbitrary feed flow rates using the same model used to simulate the permeances. Among the permeance data, the permeances simulated at a finite number of 15 on both the bore and shell sides of the membrane gave the best results in predicting the membrane performance. The differences in the CO recoveries and CO concentrations at the residue outlet between the simulated and experimental results were in the range of −1.8 to 2.0% at four feed flow rates of 5, 10, 20, and 30 L/min. The differences increased to approximately −7.3–6.9% under the same conditions when pure gas permeances were used for the simulation instead of the mixed gas permeance mentioned previously.

In this study, the NEQNF subroutine was introduced to solve the large number of nonlinear stiff equations of the model. The newly adopted solution method allows a quick calculation of the equations with an extremely small error. The mixed-flow and plug-flow membrane modules can be simulated within a few seconds with a root mean square error of less than 1.0 × 10^−16^. To our knowledge, it takes approximately one to two days to accurately simulate 2- and 3-stage membrane processes with a differential model using the trial-and-error shooting conventional solution method. Therefore, the model and solution method could be effectively exploited in multistage and large-scale membrane processes, where their mixed gas permeances can be calculated using the experimental data obtained under real operating conditions.

It is desirable to operate the LDG recovery process below 10 bars because of the high energy cost of the compressor. In the 1-stage PS membrane module with an active area of 1.0 m^2^, the achievable CO recovery with a CO_2_ concentration of less than 1% is in the range of 60–80% at this pressure condition. Moreover, a multistage process is required to enhance CO recovery. At present, a two-stage recirculation process and a mini-pilot plant have been designed and constructed using the developed model and solution technique, and are operated to separate and concentrate CO_2_ and CO from LDG.

## Figures and Tables

**Table 1:** Specifications of the PS membrane module.

Active Length (m)	ID (μm)	OD (μm)	No. of Fiber	Active Area (m^2^)
0.24	200	360	4000	1.0

**Table 2:** Gas separation results at a feed flow rate of 10 L/min, feed pressure 8 bara, and permeate pressure 1 bar.

	Flow Rate (L/min)	Concentration (%)			
		CO	CO_2_	N_2_	H_2_
Residue	6.917	74.57	4.89	20.26	0.28
Permeate	3.065	40.19	47.25	6.56	6.00

**Table 3:** Permeances and selectivities of the LDG mixed and pure gases.

Number of Finite Element			Permeance (×10^−10^ m^3^/m^2^·s·Pa)				Selectivity		
Shell	Bore	*n*	CO	CO_2_	N_2_	H_2_	CO_2_/CO	CO_2_/N_2_	CO/N_2_
10	10	1	0.3964	6.1372	0.2391	15.0102	15.66	25.96	1.66
15	15	1	0.3978	5.8752	0.2402	13.0289	14.77	24.46	1.66
20	20	1	0.3985	5.7216	0.2408	12.2034	14.36	23.76	1.65
30	30	1	0.3992	5.5757	0.2414	11.4660	13.97	23.09	1.65
15	30	2	0.3989	5.6345	0.2412	11.8931	14.13	23.36	1.65
15	45	3	0.3993	5.5574	0.2416	11.5443	14.30	23.00	1.65
15	60	4	0.3994	5.5194	0.2418	11.3750	13.82	22.83	1.65
Pure gas			0.3742	6.1522	0.2310	12.1477	16.43	26.23	1.62

**Table 4:** Experimental results at feed rates of 5, 20, and 30 L/min.

Feed Flow Rate (L/min)	Stream	Outlet Flow Rate (L/min)	Concentration (%)			
			CO	CO_2_	N_2_	H_2_
5.0	Residue	2.502	76.470	0.921	22.580	0.029
	Permeate	2.341	52.454	34.112	8.929	4.506
20.0	Residue	16.658	70.923	9.964	18.321	0.792
	Permeate	3.704	33.178	54.390	5.197	7.234
30.0	Residue	25.837	69.102	12.123	17.680	1.096
	Permeate	3.831	30.350	56.962	4.726	7.962

**Table 5:** Simulation errors with respect to the experimental data at *s* = 15 and *n* = 1 for the major performance measures of the membrane.

Feed Flow Rate (L/min)	CO Recovery (%)			CO Conc. in Residue (%)			CO_2_ Conc. in Residue (%)		
	Exper.	Simul.	Err. (%)	Exper.	Simul.	Err. (%)	Exper.	Simul.	Err. (%)
5	59.78	60.98	+2.00	76.47	76.23	−0.31	0.92	0.94	+2.17
10	80.57	80.75	+0.21	74.57	74.53	−0.05	4.89	5.07	+3.57
20	92.22	90.56	−1.80	70.92	70.97	+0.07	9.96	9.89	−0.70
30	92.99	93.77	+0.83	69.1	69.11	+0.01	12.12	12.15	+0.24
Root mean square percent error (%)			1.42			0.16			2.12

## Data Availability

Not applicable.

## Nomenclature

A	membrane area (m^2^)
C	number of gas components
D_o_	Outside diameter of membrane (m)
g	permeation rate (m^3^/s)
h	Number of bore side finite element per finite element of shell side
L	Length of membrane (m)
n	ratio of number of finite elements of bore side to shell side
Q	permeance (m^3^/m^2^.s.pa)
s	Number of shell side finite element
u	flow rate of reside side (m^3^/s)
v	flow rate of permeate side (m^3^/s)
x	concentration in residue side (volume %)
y	concentration at in permeate side (volume %)
ȳ	concentration in bulk stream of permeate side (volume %)
μ	viscosity (m^2^.pa/s)
Subscript	
f	feed
i	i component
j	j-th finite element in bore side
k	k-th finite element in shell side
m	mixed gas

## Appendix A

**Table A1:** RMSPEs calculated using the permeance of pure gases.

s	n	CO Recovery (%)	CO Conc. (%)	CO_2_ Conc. (%)
2	1	3.09	2.24	171.42
5	1	3.06	0.70	58.41
10	1	3.06	0.13	16.03
15	1	3.05	0.17	2.26
20	1	3.05	0.27	5.43
30	1	3.05	0.37	12.20
60	1	3.05	0.48	18.89

**Table A2:** CPU run times and root-mean-square errors of the NEQNF solution method.

s	Equation Number	CPU Time (sec.)	ΣF^2^	Error (Root Mean Square)
1	11	0.016	3.92 × 10^−34^	5.97 × 10^−18^
2	22	0.016	1.51 × 10^−32^	2.62 × 10^−17^
15	110	0.02	7.77 × 10^−35^	8.40 × 10^−19^
50	550	0.17	1.57 × 10^−38^	5.34 × 10^−21^
100	1100	1.62	1.59 × 10^−35^	1.20 × 10^−19^
200	2200	19.48	3.84 × 10^−35^	1.32 × 10^−19^
